# Novel Idea: Virulence-Based Therapy Against *Helicobacter pylori* Infection (Smart Therapy)

**DOI:** 10.3389/fmed.2014.00018

**Published:** 2014-06-23

**Authors:** Amin Talebi Bezmin Abadi

**Affiliations:** ^1^Department of Medical Microbiology, University Medical Center Utrecht, Utrecht, Netherlands

**Keywords:** *Helicobacter pylori*, virulence, smart therapy, novelty, treatment

## Introduction

*Helicobacter pylori* (*H. pylori*) is a Gram-negative, spiral, and microaerophilic bacterium, which can usually persist lifelong in gastric mucosa if not treated efficiently. *H. pylori* infection plays an undeniable role in the development of different gastroduodenal diseases, while its eradication cures ulcer disease and also prevents occurrence of gastric cancer (1–3). At the beginning, as usual, it was thought that antibacterial therapy could easily eliminate the infection in human gastric mucosa. As such, during the last 30 years that we have known about *H. pylori*, there have been numerous therapeutic regimens suggested (e.g., sequential, triple/dual, quadruple, DANCE, hybrid, salvage, and empirical) (4–8). Therefore, many studies have been conducted to identify the most effective and least harmful therapeutic regimen, although, a unique therapeutic regimen to cure *H. pylori* infection in all reported colonized individuals is still lacking (8, 9). However, high rates of resistance have been reported to all primary/secondary lines and even to the newly introduced alternative drugs described for *H. pylori* treatment (10). In 2014, due to the skyrocketing rates of antibiotics resistance, a new scope toward the antibiotic therapy against this mysterious bacterium seems necessary. It has been indicated that virulence factors are the ability of a bacterium to induce certain disease in attributed hosts (1, 11, 12). Accordingly, virulence factors in *H. pylori* (e.g., *cagA, dupA, homB*, and *vacA*) have essential and definite roles in pathogenesis of different gastroduodenal disorders such as chronic gastritis, gastric cancer, and peptic ulcers (13–15). Certain *H. pylori* strains (specific PCR positive for *cagA, dupA, homB*, and *vacA*) harboring virulence determinants are capable to survive longer and induce more severe diseases. Surprisingly, among the currently described studies, no therapeutic regimen according to *H. pylori* virulence pattern (virutype) has yet been suggested.

## Proposed Idea

Given the aforementioned problems in *H. pylori* treatment, it would be interesting if new therapeutic approaches can solve this complexity. Accordingly, under condition of smart therapy, we propose an idea that suggests logical application of smart therapy against *H. pylori* strains that will reduce current distribution of antibiotic resistance and also increase efficacy of prescribed antibiotics. If a clinician knows about virutype and susceptibility pattern of *H. pylori* locally, it is easy to choose a therapeutic regimen (smart therapy) that will reliably cure most of infections during the first-line therapy. In clinical settings, by prescription of smart therapy, physicians can opt to not treat non-virulent strains found in symptomatic patients, which account for more than half of all subjects. If smart therapy is validated as a new therapeutic regimen, current therapeutic failures will be considerably reduced.

## Evaluation of the Idea

In order to have a continuing effective therapeutic regimen against *H. pylori*, we practically suggest to first investigating virulence pattern (virutype) of the bacterium, therefore; antibiotic therapy should be considered only for virulent strains (smart therapy). Clinicians should only aim to detect virulent *H. pylori* according to the virulence genes, and then start to eradicate them based on local antibiotic susceptibility pattern. Indeed, detection of such virulent strains (*vacA*+, *cagA*+, *homB*+, and *dupA*+), identified by the simple sensitive PCR method, can be the main inclusion criteria to start the next step. If so, as the second step (in the case of virulent *H. pylori* strain), we need to follow an updated antibiotic susceptibility profile, which indicates the most effective drugs for each region.

## Discussion/Conclusion

*Helicobacter pylori* is inherently resistant to a few antibiotics (e.g., sulfonamides, trimethoprim, nalidixic acid, and vancomycin), and it will likely become resistant to metronidazole, clarithromycin, and fluoroquinolones, if these antibiotics are prescribed alone (16, 17). Given the significant presence of virulence factors in *H. pylori* pathogenesis and the direct link to cause more severe diseases, it is hypothesized that designation of therapeutic regimen according to virulence factors may help physicians to increase efficacy rate of therapy (18). The smart therapy strategy consists of two basic parts; (i) local antibiotic susceptibility tests and (ii) *H. pylori* virutype. The main advantage of smart therapy is its flexibility, which can give the possibility to clinicians for adjusting new antibiotics according to different localities in the world. As we already knew, antibiotic exposure is one of the main factors, which can push *H. pylori* to make new mutations, and can eventually result in more resistant genotypes. In essence, smart therapy can avoid from distribution of antibiotic resistance due to careful usage of its contained drugs for therapeutic regimens. Within smart therapy, the answer to the question of how resistant strains should be eradicated is to logically target virulent strains and smartly tackle those using effective combinations of antibiotics. We therefore conclude that our idea can enable the possible application of smart therapy in clinical practice for symptomatic digestive diseases. According to the smart therapy, more studies of prescribed drugs and certain virulence genes are suggested to examine their potential to be incorporated as virutypes of *H. pylori*.

## Conflict of Interest Statement

The author declares that the research was conducted in the absence of any commercial or financial relationships that could be construed as a potential conflict of interest.
